# Regulatory T Cells in Human Ovarian Cancer

**DOI:** 10.1155/2012/345164

**Published:** 2012-02-06

**Authors:** Dong-Jun Peng, Rebecca Liu, Weiping Zou

**Affiliations:** ^1^Department of Surgery, University of Michigan, Ann Arbor, MI 48109, USA; ^2^Department of Obstetrics and Gynecology, University of Michigan, Ann Arbor, MI 48109, USA; ^3^Graduate Programs in Immunology and Cancer Biology, University of Michigan, Ann Arbor, MI 48109, USA; ^4^University of Michigan School of Medicine, C560B MSRB II, 1150 W. Medical Center Dr., Ann Arbor, MI 48109-0669, USA

## Abstract

Multiple layers of suppressive components including regulatory T (T_Reg_) cells, suppressive antigen-presenting cells, and inhibitory cytokines form suppressive networks in the ovarian cancer microenvironment. It has been demonstrated that as a major suppressive element, T_Reg_ cells infiltrate tumor, interact with several types of immune cells, and mediate immune suppression through different molecular and cellular mechanisms. In this paper, we focus on human ovarian cancer and will discuss the nature of T_Reg_ cells including their subsets, trafficking, expansion, and function. We will briefly review the development of manipulation of T_Reg_ cells in preclinical and clinical settings.

## 1. Introduction

Ovarian cancer is one of the most common and deadliest gynecologic cancers. In 2010, 21880 new cases were diagnosed, and such cancer caused nearly 13850 deaths in the United States alone [1]. Ovarian cancer usually has poor prognosis, and most patients were diagnosed at advanced stages. The five-year survival rate for all stages of ovarian cancer is 46% in 2010 [1]. It has been well documented that patients' clinical outcome and five-year survival rate are positively associated with the number of tumor-infiltrating lymphocytes (TILs) [2], and the ratio of intraepithelial CD8^+^ TILs to T_Reg_ cells [3], or negatively associated with tumor- infiltrating T_Reg_ cells [4].

T_Reg_ cells are also known as suppressor T cells which consist of a specific subpopulation of cells that functionally suppress the activation of immune system and maintain immune tolerance to self-antigens. T_Reg_ cells contain two major subsets known as natural T_Reg_ cells (nT_Reg_) and adaptive or induced T_Reg_ cells (iT_Reg_). nT_Reg_ cells derived from thymus are considered as classic T_Reg_ cells, by contrast, iT_Reg_ cells develop in the periphery in response to self- or tumor antigens by converting naive CD4^+^ T cells into T_Reg_ cells [5]. Because most tumors express self-antigens, T_Reg_ cells-mediated immunosuppression is believed to be one of the major contributors to immune evasion by tumors and becomes the main obstacle toward successful tumor immunotherapy [6]. In this paper, we will focus on human ovarian cancer and discuss the nature of T_Reg_ cells including their subsets, trafficking, differentiation, and proliferation and the clinical application of manipulation of T_Reg_ cells.

## 2. Regulatory T-Cell Subsets

In early 1970s, Gershon and Kondo first described the existence of thymus-derived suppressive T cells (later termed as T_Reg_ cell) *in vivo* [7, 8]. After more than a decade, Sakaguchi et al. demonstrated that CD4^+^ T cells expressing interleukin-2 (IL-2) receptor alpha-chain (CD25) can be defined as the population of T_Reg_ cells with immune-suppressive activities and maintaining immune tolerance to self-antigen [9]. Later in 2003, Hori et al. found that the transcription factor forkhead box P3 (Foxp3) controls the development of T_Reg_ cells and is crucial for maintaining the immune-suppressive function of T_Reg_ cells [10].

Natural T_Reg_ cells differentiate in the thymus and migrate to periphery, which constitute 5–10% of CD4^+^ T cells [11–13]. In addition, there are several subsets of T_Reg_ cells other than CD4^+^CD25^+^Foxp3^+^  T_Reg_ cells. Groux et al. identified another subset of T_Reg_ cells, CD4^+^  T_R_ 1 cells, that suppress antigen-specific immune responses by producing high levels of IL-10 [14]. In addition to CD4^+^  T_Reg_, CD8^+^ suppressive T cells have been found playing an important role in the regulation of autoimmune disease [7, 15]. CD8^+^ suppressive T cells now refered to as CD8^+^  T_Reg_ cells are characterized as CD8^+^CD25^+^, CD8^+^CD122^+^, or CD8^+^CD45RC^low^  T_Reg_ cells, which comprise less than 1% of peripheral CD8^+^ T cells [15]. Th3 T_Reg_ cells have similar immune-suppressive function; however, in contrast to natural T_Reg_ cells, Th3 exerts its suppressive capacity independent of cell membrane contact but mainly bases on the action of self-produced cytokine TGF*β* [16].

## 3. Regulatory T-Cell Trafficking

T_Reg_ cells consist of ~10% of peripheral CD4^+^ T cells characterized as CD4^+^CD25^+^FOXP3^+^ T cells, which is important for the control of autoimmune reaction [9, 11]. Dysregulation of T_Reg_ can cause autoimmune diseases [17] and may contribute to tumor-initiated immune evasion [18]. As demonstrated by *in vivo* mouse model, the deletion of T_Reg_ cells results in tumor rejection [19]. However, the suppressive capacity of T_Reg_ cells is also determined by the ratio of T_Reg_ cells to effector T cells [3]. A high CD8^+^/T_Reg_ ratio is associated with favorable prognosis and improved survival [3, 20]. It has been reported that many human cancers are associated with high frequency of T_Reg_ cells in the circulation or in the tumor tissues, including ovarian cancer [4], lung cancer [21], breast cancer [22], liver cancer [23], head and neck cancer [24], and lymphoma [25]. These increased levels of T_Reg_ cells are linked to high death hazard and poor survival, while the depletion of tumor-infiltrated T_Reg_ cells and the blockade of T_Reg_ trafficking to tumors enhance anti-tumor immune response [4, 26].

CCR4 and its binding partners CCL22 and CCL17 are believed to be the most predominant axis in chemokine-mediated selective T_Reg_ trafficking to the tumors. Iellem et al. have profiled chemotactic responses and chemokine receptors expression of human T_Reg_ cells and found that T_Reg_ cells specifically express chemokine receptors CCR4 and CCR8 [27]. Chemokine CCL22, the ligand for CCR4, preferentially attracts activated-antigen-specific T cells to dendritic cells [28, 29]. It has also been shown that human ovarian cancer cells and tumor-associated microphages produce chemokine CCL22, which mediates T_Reg_ cells trafficking to tumor [4]. Blockade of CCL22 *in vivo* significantly reduces human T_Reg_ cells trafficking to tumors in ovarian carcinoma [4]. This chemokine-mediated T_Reg_ trafficking has been also observed in other types of cancer, such as gastric cancer [30], Hodgkin's lymphoma [31], and breast cancer [32]. Interestingly, in gastric cancer, CCL22 and CCL17 seem both important to recruit T_Reg_ cells to the tumors as demonstrated by *in vivo* study as well as *in vitro* migration assay, and the levels of CCL22 and CCL17 within tumors are correlated to the increased levels of T_Reg_ cells in early gastric cancer [33].

Besides CCR4 chemokine axis, CCR5/CCL5 axis may also selectively recruit T_Reg_ cells to the tumors. Using human pancreatic adenocarcinoma and murine pancreatic tumor model, it has been found that CCR5 is highly expressed in T_Reg_ cells, while tumor cells produce elevated amount of CCL5, and disruption of CCR5/CCL5 chemokine axis blocks T_Reg_ cells migration and reduces tumor growth [34]. In addition, CCL20 chemokine shows high affinity to CCR6 and can also mediate selective CCR6^+^ T_Reg_ cells trafficking [35].

## 4. Regulatory T-Cell Differentiation and Proliferation

CD4^+^CD25^+^  T_Reg_ cells are generated in the thymus. Papiernik et al. found that peripheral T_Reg_ migrates from the thymus and appears in the periphery as early as 10th day of life [36]. They also found that CD4^+^CD25^+^  T_Reg_ cells differentiation is totally dependent on IL-2, because IL-2 knockout mice do not develop CD4^+^CD25^+^  T_Reg_ *in vivo* [36]. Further evidences have been provided from the studies on irradiated rat model [37]. In this study, autoimmune diseases were induced in rats by thymectomy and irradiation; however the xenograft transfer of CD4^+^ T cells from normal rats can abrogate the autoimmune responses. These observations suggest that normal thymus-derived T cells have immune suppressive functions and thus prevent autoimmunity [37]. In another model system, adoptive transfer of thymocytes or peripheral T cells depleted of CD4^+^CD25^+^  T_Reg_ cells causes autoimmune diseases in mice, which provides further evidences of thymic origin of T_Reg_ cells and their peripheral existence [38].

However, there is little known about the comprehensive requirements for thymic T_Reg_ development. Although there are several arguments about how and what stromal components are involved in thymic T_Reg_ cell differentiation, thymic stromal cells, including cortical and medullary thymic epithelial cells and dendritic cells (DCs), contribute to T_Reg_ cells differentiation and selection [38]. Jordan et al. used TCR-transgenic mice which express the receptor recognizing specific self-antigen and found that thymocytes bearing a TCR with high affinity to a specific self-antigen undergo selection and become CD4^+^CD25^+^  T_Reg_ cells when interacting with a single self-antigen, but thymocytes bearing TCR with low affinity do not undergo selection [39].

In addition to thymus, T_Reg_ can also be generated in the periphery. For instance, tumor microenvironment favors the induction and differentiation of T_Reg_ cells, and that has been extensively studied for several years [40]. In the tumor microenvironment, DC differentiation and function were suppressed by tumor-associated factors IL-10, VEGF, and TGF*β*, resulting in immature/dysfunctional DC [6]. Dysfunctional DC directly contributed to the induction of IL-10-producing T_Reg_ cells *in vivo* in human and *in vitro* [41, 42]. Tumor-associated plasmacytoid DC also induced IL-10^+^  T_Reg_ generation [43, 44]. Tumor can convert DC into TGF*β*-producing immature DC, which selectively promotes T_Reg_ proliferation in TGF*β*-dependent manner [45].

CD4^+^CD25^+^  T_Reg_ cells can also be converted from peripheral naïve CD4^+^CD25^−^ T cells by the action of TGF*β*. Tumor microenvironment contains high levels of TGF*β* which might mediate tumor-associated T_Reg_ cells conversion [46].

## 5. Targeting Regulatory T Cells

### 5.1. T_Reg_ Cell Depletion

In the mouse model, depletion of CD4^+^CD25^+^  T_Reg_ cells using anti-CD25 antibody causes tumor regression, which correlated to the reduced number of T_Reg_ cells [18, 47]. Using the recombinant IL-2 diphtheria toxin conjugate DAB(389)IL-2 (also known as denileukin diftitox and ONTAK), Dannull et al. demonstrated that DAB(389)IL-2 was capable of selectively eliminating CD25^+^  T_Reg_ cells from the PBMCs of cancer patients without inducing toxicity on other cellular subsets, and DAB(389)IL-2-mediated T_Reg_ depletion enhanced anti-tumor immune responses and significantly reduced the number of T_Reg_ cells present in the blood of cancer patients [48]. Daclizumab (also known as Zenapex) and Basiliximab (also called Simulect) are monoclonal antibodies against CD25 [49, 50], and the administration of Daclizumab in patient with metastatic breast cancer enhanced anti-tumor immunity [51].

Cytotoxic T-lymphocyte-associated antigen 4 (CTLA4) is constitutively expressed and restricted to CD4^+^CD25^+^  T_Reg_ cells among all CD4^+^ cells, and the immune-suppressive function of T_Reg_ is mediated by CTLA4 signaling [52, 53]. CTLA4 binds to inhibitory B7 members on APC and transmits an inhibitory signal to T cells. *In vivo* administration of anti-CTLA4 antibody resulted in tumor rejection including preestablished tumors [54]. Periodic infusions of anti-CTLA4 antibody in previously vaccinated patients with cancer created clinically effective antitumor immune response [55]. Patients with metastatic melanoma showed improved antitumor immunity and tumor regression by blockade of CTLA-4 together with peptide vaccination [56].

Glucocorticoid-induced tumor necrosis factor (TNF) receptor family-related protein (GITR or DTA-1) is predominantly expressed on the surface of T_Reg_ cells. An agonistic anti-GITR antibody administration in mice can abrogate T_Reg_-mediated immune suppression and enhance effective anti-tumor immunity *in vivo* [57, 58]. In addition, treatment with anti-GITR antibody in B16 mice elicited immune response and rejected tumor [59]. However GITR is not exclusively expressed on T_Reg_ cell; it is also expressed by various CD4^+^ T cells and others. Therefore, the clinical therapeutic relevance of GITR blockade and its side effects on potential deficits of other effective immune cells remain to be determined.

OX40 (CD134) also belongs to TNF receptor family and expressed on activated T cells. Both naïve and activated T_Reg_ express OX40. Similar to GITR, triggering OX40 by an agonistic antibody against OX40 reduces T_Reg_-mediated immune suppression and restores effector T-cell function both *in vivo* and *in vitro* [60]. It has been also shown that OX40 is necessary for T_Reg_ development, homeostasis, and immune-suppressive activity. However, stimulation of OX40 signal in naïve T cells can abrogate T_Reg_-mediated suppression [61].

Clinical relevance of the depletion of T_Reg_ cells has been further confirmed by the treatment of cyclophosphamide (CY) in the patients bearing tumor. Cyclophosphamide is a nitrogen mustard alkylating agent that mediates DNA crosslinking. Low dose of CY administration improved patients' immune responses by reducing the number of T_Reg_cells and by decreasing the suppressive activity of T_Reg_ cells [62]. Effects of T_Reg_ depletion on anti-tumor immune responses were further investigated by the study on B16 melanomas mouse model [63]. Other immunosuppressants like cyclosporine A (CSA) and azathioprine might also inhibit T_Reg_ cells generation [64, 65]. For instance, high dose of CSA abrogates T_Reg_ cell generation; by contrast, low dose of CSA can promote T_Reg_ cell development [64]. It is therefore important to determine whether lowdose of those agents can improve antitumor immunity in patients.

### 5.2. Targeting T_Reg_ Trafficking

Our group has demonstrated that human ovarian cancer cells and tumor-associated macrophage (TAM) produced chemokine CCL22, the ligand for CCR4 which functionally expressed on tumor T_Reg_ cells, mediating T_Reg_ cells trafficking to the tumor and ascites, and the blockade of CCL22 abrogated T_Reg_ cells migration [4]. It has been demonstrated that chemokine receptor CCR4 is selectively expressed by T_Reg_ cells, and the CCR4 and CCR4-associate chemokines axis is one of the most described tumor T_Reg_ recruitment axes [66]. The administration of anti-CCR4 antibody effectively depletes CCR4^+^ T cells and inhibits T_Reg_ cells migration in Hodgkin lymphoma [31]. Furthermore, the significant correlation between CCL17 or CCL22 chemokines and the number of tumor-infiltrating T_Reg_ cells was found in patients with neoplastic meningitis and gastric cancer [30, 33]. CCL5 and CCL20 chemokines are also involved in T_Reg_ trafficking, and that blockade of those chemokines reduces T_Reg_ cells trafficking and inhibits tumor growth [34, 35]. We have shown that CXCL12/CXCR4 axis mediated T_Reg_ trafficking to bone marrow [67]. Recently, a study has demonstrated that blockade of CXCR4 by a selective antagonist resulted in the significant reduction of intratumoral T_Reg_ cells, which was associated with greatly increased antitumor immunity and an improved survival in an immunocompetent mouse model of ovarian cancer [68].

### 5.3. Targeting TGF*β* Signaling Pathway

TGF*β* is implicated in T_Reg_ differentiation, conversion, and function. It is thought that blockade of TGF*β* signaling pathway may alter T_Reg_ phenotype and function and in turn enhances antitumor immunity [6]. In addition to T_Reg_ cells, ovarian carcinoma cells can also produce TGF*β* [69]. Notably, TGF*β* is not only important for T_Reg_ cell functional integrity, but also inhibits the proliferation and functional differentiation of T lymphocytes, NK cells, and macrophages [46, 70]. This may induce T-cell unresponsiveness to TCR stimulation, failure to produce Th1 cytokines, and production of additional TGF*β* [46]. TGF*β* signaling may also be crucial for tumor cell transformation. Therefore, targeting TGF*β* signaling may be therapeutically meaningful. TGF*β* inhibitor AP 12009 was tested in a Phase I/II clinical trial for advanced pancreatic cancer and other malignancies [71]. LY2109761, an inhibitor of TGF*β* I/II receptors, can suppress pancreatic cancer metastases [72]. In a preclinical model, we have shown that anti-TGF*β* can reduce T_Reg_ cells in tumors and tumor-draining lymph nodes. This effect is enhanced by B7-H1 blockade [73]. Nonetheless, it is clear that blocking TGF*β* signaling may affect T_Reg_ compartment. However, as TGF*β* is implicated in multiple layers of biological activities, the ultimate clinical therapeutic efficiency and side effects of TGF*β* signaling blockade remain to be investigated.

### 5.4. Targeting Inhibitory B7 Family Members

The expression, regulation, functional, and clinical relevance of inhibitory B7 family members have been reviewed elsewhere [74]. Human ovarian cancer and cancer-associated myeloid antigen-presenting cells express high levels of B7-H1 (PD-L1), which are negatively associated with patient survival [74, 75]. Patients with high expression of B7-H1 had a significantly poor prognosis compared to the patients with low expression of B7H1 [76]. B7-H1 expression was also found inversely correlated to the intraepithelial CD8^+^ T lymphocyte count, indicating that B7-H1 on tumor cells may suppress antitumor CD8^+^ T cells [76]. The receptor, programmed death 1 (PD-1), is expressed on activated T-cell subsets, antigen-specific CD8^+^ T cells [77], and T_Reg_ [78]. Interestingly, B7-H1/PD-1 has been reported to be involved in the development of induced T_Reg_ cells [79]. Therefore, targeting B7-H1/PD-1 signaling pathway may reduce T_Reg_ development and function. As anti-PD-1 is in clinical application to treat patients with melanoma, renal cell carcinoma, and other cancers, further mechanistic studies on these patients will determine if the effects of anti-PD-1 on T_Reg_ cells are mechanistically and clinically relevant.

In addition to B7-H1, human ovarian cancer and cancer-associated myeloid antigen-presenting cells also express high levels of B7-H4 (B7x, B7s1), which are negatively associated with patient survival [74, 80, 81]. Interestingly, T_Reg_ cells can induce IL-10 expression by APCs and indirectly stimulate B7-H4 expression on APCs and convey suppressive activity to APCs [74, 80, 81]. Thus, it is tempting to speculate that blocking B7-H4 signaling pathway may disable the suppressive effects of T_Reg_ cells on APCs. Notably, as the receptor for B7-H4 has not been identified, B7-H4 signaling is much less understood in both mouse and human system. Nonetheless, studies on ovarian cancer patients and preclinical cancer models suggest that interruption of B7-H4 signaling may lead to improved antitumor T-cell response and decreased T_Reg_ suppressive function.

## 6. Conclusions

T_Reg_ cells infiltrate tumor including ovarian cancer. Their phenotype, trafficking mechanism, suppressive activity, and clinical relevance have been defined in human cancer. However, recent evidence indicates that T_Reg_ cells may not be stable and are subject to environmental regulation. In this regard, it remains poorly understood how T_Reg_ cells evolve in human tumor microenvironment. Although their action mode of mechanisms has been investigated in many different physiological and pathological scenarios, the key suppressive mechanisms may be differed in different tumors or/and in different stages. Therefore, further patient-oriented studies are essential for dissecting T_Reg_ cell biology. Nonetheless, targeting T_Reg_ cells or/and reprogramming T_Reg_ cells is an important strategy to treat patients with cancer. It is suggested that combinatorial therapy by incorporating T_Reg_ manipulation may be ideal direction to develop novel therapeutic regimen to efficiently treat patients with cancer.
